# Public Support for Vehicle Technology to Prevent Operation by Impaired Drivers

**DOI:** 10.1001/jamanetworkopen.2023.9152

**Published:** 2023-04-20

**Authors:** Johnathon P. Ehsani, Jeffrey P. Michael, Shannon Frattaroli, Gayane Yenokyan, Ahmed Sabit

**Affiliations:** 1 Department of Health Policy and Management, Johns Hopkins Bloomberg School of Public Health, Baltimore, Maryland; 2 Johns Hopkins Biostatistics Center, Department of Biostatistics, Johns Hopkins University Bloomberg School of Public Health, Baltimore, Maryland

## Abstract

This survey study measures public support for vehicle impairment prevention technology in the US.

## Introduction

Motor vehicle crash deaths are increasing at a historic rate, and alcohol is associated with close to one-third of all fatalities.^1^ Vehicle impairment prevention technologies can recognize whether a driver is dangerously impaired by alcohol or other causes and prevent an impaired driver from operating the vehicle. Installed in every vehicle, this technology could save more than 9000 lives per year.^2^ The Infrastructure Investment and Jobs Act of 2021 mandates that by November 2024, the US Department of Transportation establish a Federal Motor Vehicle Safety Standard requiring impairment prevention systems in all new cars within 3 subsequent years.^3^ Public support for impairment prevention technologies is not thoroughly understood. We conducted a national survey measuring public support for this technology, and how this compares to a range of other safety technologies.

## Methods

The survey was fielded from May 4 to June 10, 2022, using the probability-based AmeriSpeak Panel (NORC), designed to represent the US adult population.^4^ The sample was drawn from this panel and administered via telephone and online, in English and Spanish. NORC obtains informed consent prior to enrolling individuals in the panel. The Johns Hopkins Bloomberg School of Public Health Institutional Review Board deemed this study exempt from review because it did not constitute human participant research. The study followed the AAPOR reporting guideline.

First, we measured the support for the federal mandate for vehicle impairment prevention technology using a 5-point scale. In addition, support for a range of vehicle safety technologies was measured by asking whether respondents agreed that all new vehicles should have speed limiters, cell phone blockers, speed warnings, seat belt interlocks, fatigue warnings, and vehicle impairment prevention. A variable for overall support or agreement and overall opposition or disagreement was created by combining responses of strongly and somewhat (details are provided in the eMethods in Supplement 1). Prevalence estimates and their 95% CIs incorporated sampling weights to generate nationally representative estimates. Analyses were conducted using R, version 4.2.2 (R Project for Statistical Computing).

## Results

The survey completion rate was 31.6%, with a final sample of 2274 adults aged 18 years or older (1384 [60.9%] women and 890 [39.1%] men). Respondents had a mean (SD) age of 44.3 (14.3) years. Support for the congressional mandate on vehicle impairment prevention technology was high, with 63.4% (95% CI, 61.3%-65.5%) of respondents supporting the law that had passed. Speed limiters (30.7% [95% CI, 28.6%-32.7%]) and cell phone blocking (37.4% [95% CI, 35.3%-39.45%]) were the least popular technologies. Speed warnings (53.1% [95% CI, 51.1%-55.2%]) and seat belt interlocks (57.1% [95% CI, 55.0%-59.2%]) were substantially more popular. Technologies with the highest proportion of support were vehicle impairment prevention (64.9% [95% CI%, 62.8%-66.9%]) and fatigue warnings (77.3% [95% CI, 75.2%-79.3%]) (Figure).

**Figure.**
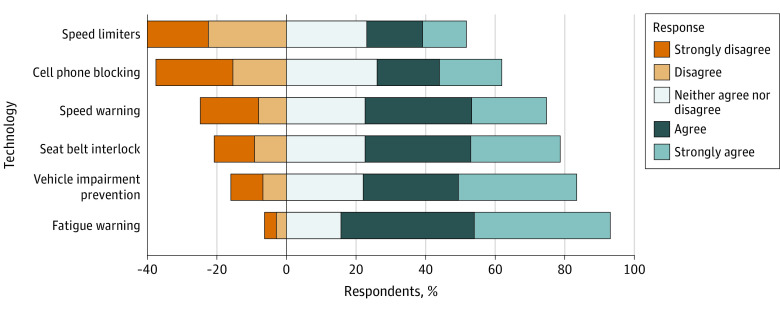
Survey Results Respondents were asked whether the specified safety technology should be standard in all new vehicles.

## Discussion

In this nationally representative survey, most US adults were in favor of the recent congressional action related to vehicle impairment prevention technology. There was variability in agreement with statements about the types of safety technologies all new vehicles should have, which indicates that public acceptance of safety technologies depends on specific purposes. In the case of impairment prevention, most respondents agreed that this feature should be standard in all new vehicles. Widespread public endorsement suggests that social norms rejecting impaired driving are well established^5^ and the technology will be broadly accepted. Automakers and regulators can advance with implementation of the mandate with the expectation that most of the public will support the change. While NORC used probability-based recruitment consistent with best practices for survey research, these results may be vulnerable to sampling biases.

## Notes

Supplement 1. **eMethods.** Survey Questions and Survey Methods Used by NORC
eReferences

Supplement 2.
Data Sharing Statement
