# Supplementary material for: Public Support for Vehicle Technology to Prevent Operation by Impaired Drivers
Source: JAMA Netw Open. 2023 Apr 20;6(4):e239152. doi: 10.1001/jamanetworkopen.2023.9152 (PMC10119736; doi:10.1001/jamanetworkopen.2023.9152)
Supplement: Supplement 1. — eMethods. Survey Questions and Survey Methods Used by NORC eReferences [file jamanetwopen-e239152-s001.pdf]

Supplementary Online Content

Ehsani JP, Michael JP, Frattaroli S, Yenokyan G, Sabit A. Public support for vehicle technology to prevent operation by impaired drivers. *JAMA Netw Open*. 2023;6(4):e239152. doi:10.1001/jamanetworkopen.2023.9152

eMethods. Survey Questions and Survey Methods Used by NORC
eReferences.

This supplementary material has been provided by the authors to give readers additional information about their work.

© 2023 Ehsani JP et al. *JAMA Network Open*.

eMethods. Survey Questions and Survey Methods Used by NORC

Survey questions with response options

1. **Do you support or oppose the recent action by Congress to require drunk driving prevention technology in all new vehicles?**

RESPONSE OPTIONS:

A. Strongly support
B. Somewhat support
C. Somewhat oppose
D. Strongly oppose

2. **Please indicate your level of agreement or disagreement with the following statements:**

GRID ITEMS, RANDOMIZED:

a. All new cars should have a warning device to alert drivers if they are falling asleep when driving.
b. All new cars should have a warning device to alert drivers if they are driving 10mph over the speed limit.
c. All new cars should have automatic speed control so they cannot go more than 10mph over the speed limit.
d. All new cars should have an automatic sensor to prevent the car from being driven by someone who is over the legal alcohol limit.
e. All new cars should have seat belt sensors that would prevent the car from being driven if the driver or passengers are not wearing a seat belt or in a child safety seat.
f. All new cars should have cell phone blocking technology that does not allow the driver to talk on the phone, text, or browse the internet while driving.

RESPONSE OPTIONS:

A. Strongly Agree
B. Agree
C. Neither Agree nor Disagree
D. Disagree
E. Strongly Disagree

© 2023 Ehsani JP et al. *JAMA Network Open*.

Survey methods used by NORC

The sampling approach and weighting plan used by NORC are described below.

Sampling

The general population of the survey sample (age 18+) was selected from NORC's AmeriSpeak Panel using sampling strata based on age, race/Hispanic ethnicity, education, and gender (48 sampling strata in total). Sample selection accounted for the expected differential survey completion rates across the sampling strata. The size of each stratum's selected sample was chosen to ensure the distribution of complete surveys across the strata matched that of the target population as represented by census data. If a panel household had more than one active adult panel member, only one adult panel member was selected into the sample. When panelists were selected for an AmeriSpeak survey, the selection process within each sampling strata favored those who were not selected in the most recent AmeriSpeak survey. This selection process was designed to minimize the number of surveys any one panelist was exposed to and maximize the rotation of all panelists across AmeriSpeak surveys.[1]

Statistical Weighting

The final weight variables that were applied to the data are a product of three weights:

AmeriSpeak Panel Weights: Weights developed for all panel members to account for their probability of selection into the sample of panel recruits, panel recruitment nonresponse adjustments, and poststratification adjustments of the recruited panel to match population benchmarks.

Study Specific Base Weights: Sampling weights developed for a study sample selected from the panel to account for their selection probabilities under the sample design. The base weights were a product of the AmeriSpeak Panel Weights and the inverse of selection probabilities associated with sample selection from the panel.

Study Specific Final Weights: These were final weights developed for all competed cases of a specific study. The final weights were adjustments of the base weights to address survey nonresponse through a weighting class method. Raking adjustment were then applied to the non-response adjusted weights to align the survey sample to specific population benchmarks. The final weights may have been trimmed to reduce the influence of extreme weights on survey estimates.

For Adults Age 18+

AmeriSpeak Panel Weights**:** Since the sampling frame for this study was the AmeriSpeak Panel, which itself was a sample, the starting point of the weighting process for the study was the AmeriSpeak panel weight. To develop the panel weight, NORC first computed the panel base weight as the inverse of the probability of selection from the NORC National Frame (the sampling frame that is used to sample housing units for AmeriSpeak) or other address-based sample frames (supplemental panel samples were selected from frames developed from the USPS Delivery Sequence Files). The sample design and recruitment protocol for the AmeriSpeak Panel involved unequal sampling rates across the sampling strata and additional subsampling of initial nonresponding housing units for in-person nonresponse follow-up (NRFU). The panel base weights reflected all the variations in panel sample selection probabilities. The panel base weights were then adjusted to account for unknown eligibility and nonresponse among eligible housing units. These adjustments were conducted using weighting classes defined by some household characteristics provided by commercial data vendors, including partisan score, political party identification, the presence of young adult(s), and minority status. To produce the

© 2023 Ehsani JP et al. *JAMA Network Open*.

final household panel weights, the household-level nonresponse adjusted weights were post-stratified to match the number of households per census division obtained from the most recent Current Population Survey (CPS). Final household weights were assigned to each eligible adult in the recruited household. These person-level weights were then adjusted to compensate for nonresponding adults within a recruited household. Finally, the nonresponse adjusted person-level panel weights were raked to population totals associated with the following variables:

Variables & the Variable Categories for Panel Recruitment Non-Response Raking
Age: 18-24, 25-29, 20-39, 40-49, 50-59, 60-64, and 65+
Gender: Male and Female
Census Division: New England, Middle Atlantic, East North Central, West North Central, South Atlantic, East South Central, West South Central, Mountain, and Pacific
Race/Ethnicity: Non-Hispanic White, Non-Hispanic Black, Hispanic, Non-Hispanic Asian or Pacific Islander, and Non-Hispanic Other
Education: Less than High School, High School/GED, Some College, and BA and Above
Housing Tenure: Homeowner and Other
Household phone status: Cell Phone-only, Dual User, and Landline-only/Phoneless
Age x Gender: 18-34 Male, 18-34 Female, 35-49 Male, 35-49 Female, 50-64 Male, 50-64 Female, 65+ Male, and 65+ Female
Age x Race/Ethnicity: 18-34 Non-Hispanic White, 18-34 All Other, 35-49 Non-Hispanic White, 35-49 All Other, 50-64 Non-Hispanic White, 50-64 All Other, 65+ Non-Hispanic White, and 65+ All Other

The external population totals were obtained from the Current Population Survey, except for Household Phone Status which was determined using the National Institutes of Health bi-annual survey on wireless substitutions.[2] The weights adjusted to the external population totals were the *final panel weights*.

Study Specific Base Weights: These were developed to adjust for unequal selection probabilities from the AmeriSpeak panel, differential nonresponse across subpopulations, and frame coverage limitations. All these weighting adjustments were applied to the final panel weights described above.

The sample for this study was selected from the AmeriSpeak Panel using sampling strata (see the description of the sampling strata for this study earlier in this report). Sample selection considered the expected differential survey completion rates across these strata based on average completion rates in previous surveys. This sample selection based on expected nonresponse ensured a more representative final sample of completed interviews. However, the net result of the sampling design was an unequal selection probability that varied depending on the strata a respondent represented. For this specific study, the strata used to calculate the probability of selection included the 48 sampling strata referenced above as well as whether a panelist was a likely parent of a child under the age of 13. *Study-specific base weights* were computed as the product of the final panel weights and this calculated inverse of the probabilities of selection.

Finally, ***Study Specific Final Weights*** were created by first adjusting the base weights for survey nonresponse through a weighting class method, where the weighting classes were defined by age, race/ethnicity, gender, and education. After that, a raking ratio adjustment was applied to the nonresponse adjusted base weights to align the sample with known population benchmarks across the entire 18+ sample made up of the topline socio-demographic characteristics of the following:

Variables & the Variable Categories for Study-Specific Survey Non-Response Raking
Age: 18-24, 25-29, 20-39, 40-49, 50-59, 60-64, and 65+
Gender: Male and Female

Census Division: New England, Middle Atlantic, East North Central, West North Central, South Atlantic, East South Central, West South Central, Mountain, and Pacific
Race/Ethnicity: Non-Hispanic White, Non-Hispanic Black, Hispanic, and Non-Hispanic Other
Education: Less than High School, High School/GED, Some College, and BA and Above
Age x Gender: 18-34 Male, 18-34 Female, 35-49 Male, 35-49 Female, 50-64 Male, 50-64 Female, 65+ Male, and 65+ Female
Age x Race/Ethnicity: 18-34 Non-Hispanic White, 18-34 All Other, 35-49 Non-Hispanic White, 35-49 All Other, 50-64 Non-Hispanic White, 50-64 All Other, 65+ Non-Hispanic White, and 65+ All Other
Race/Ethnicity x Gender: Non-Hispanic White Male, Non-Hispanic White Female, All Other Male, and All Other Female

© 2023 Ehsani JP et al. *JAMA Network Open*.

eReferences

1. Dennis M. *Technical Overview of the AmeriSpeak® Panel: NORC's Probability-Based Research Panel*. Univeristy of Chicago; 2019.

2. Blumberg SJ, Luke JV. *Wireless Substitution: Early Release of Estimates From the National Health Interview Survey, January-June 2021*. National Center for Health Statistics; 2021.

© 2023 Ehsani JP et al. *JAMA Network Open*.
