# Supplementary material for: Public Support for Vehicle Technology to Prevent Operation by Impaired Drivers
Source: JAMA Netw Open. 2023 Apr 20;6(4):e239152. doi: 10.1001/jamanetworkopen.2023.9152 (PMC10119736; doi:10.1001/jamanetworkopen.2023.9152)
Supplement: Supplement 2. — Data Sharing Statement [file jamanetwopen-e239152-s002.pdf]

Data Sharing Statement

Ehsani. Public Support for Vehicle Technology to Prevent Operation by Impaired Drivers. *JAMA Netw Open*. Published April 20, 2023. doi:10.1001/jamanetworkopen.2023.9152

Data
Data available: No
